# Microtremor data and HVSR method in the kaligarang fault zone Semarang, Indonesia

**DOI:** 10.1016/j.dib.2023.109428

**Published:** 2023-07-17

**Authors:** Tony Yulianto, Gatot Yuliyanto

**Affiliations:** Department of Physics, Faculty of Science and Mathematics, Diponegoro University, Prof. Soedarto Street No. 50275, Tembalang, Tembalang District, Semarang City, Central Java 50275, Indonesia

**Keywords:** Seismic data, P-wave, S-wave, Density, Hv curve

## Abstract

The data presented contains a collection of microtremor measurement data in the Kaligarang Fault zone, Indonesia. This study aims to present data regarding the structure and lithology of the rocks in the Kaligarang Fault zone. Data was obtained using a triaxial geophone VHL PS 2B and recorded with a data logger type GL 240 with a duration of 10 minutes. The data in this study are primary data taken using a single-station microtremor device with a total of 75 acquisition points in the Kaligarang Fault zone. This data can then be used in the analysis of the Horizontal to Vertical Spectral Ratio (HVSR) which will later produce the hv curve. The v_p_, v_s_, density and thickness profiles of each data were obtained from carrying out the inversion process using dinver.

**Specifications Table**

**Table:** 

Subject	Geophysics, Physics, Earth Sciences
Specific subject area	Earthquake Engineering, Near-surface geophysics, Tectonic Geomorphology
Type of data	Excell, Figure, txt, .model
How the data were acquired	Data acquisition was carried out by measuring microtremor signals using a triaxial geophone VHL PS 2B and recording with a data logger type GL 240.
Data format	Raw, Filtered, Inversion
Description of data collection	Field conditions and coordinates were recorded during raw data measurement. The potential for movement or disturbance around the seismogram is also recorded as noise in the data. The seismometer must be checked first so that it is integrated with the data logger. Placement of the seismometer must be in an area that has stable soil, cleared of gravel, grass and roots. Make sure the bubbles on the seismometer are right in the middle of the bull's eye level and the direction of the seismogram is pointing north. In this paper, 10 minutes of microtremor data is considered sufficient to represent each location.
Data source location	City/Town/Region: Semarang, Central Java Country: Indonesia Latitude and longitude samples/data: -7.1053, 110.4099
Data accessibility	https://data.mendeley.com/datasets/3y496txbc6
Related research article	N/A

## Value of the Data

• Microtremor data can be used to improve further testing on other fault zone cases.
• The microtremor data set can be used as a comparison and linked to other geophysical measurement tools or methods
• The data allows for analysis of lithology and subsurface structures, such as stratification within geological units, and faults.
• Provide information related to fault zones to the local government for consideration of urban planning for the area around the fault zone.
• Raw data can be reprocessed so that it can display 3D images in the Kaligarang fault zone

## 1 Objective

This data set is designed to investigate research on rock structures in fault zone areas. The knowledge gained by conducting this research can be used to determine fault structure, seismic vulnerability, amplification factors etc. The data set is measured over a fairly large range of locations. Having sufficient and extensive data can avoid excessive data extrapolation so that the conditions below the ground surface can be thoroughly, broadly and accurately known along the Kaligarang fault zone, Indonesia. The existence of research on this fault zone can be additional data on the Kaligarang fault zone, Indonesia.

## 2 Data Description

**Fig. 1:**
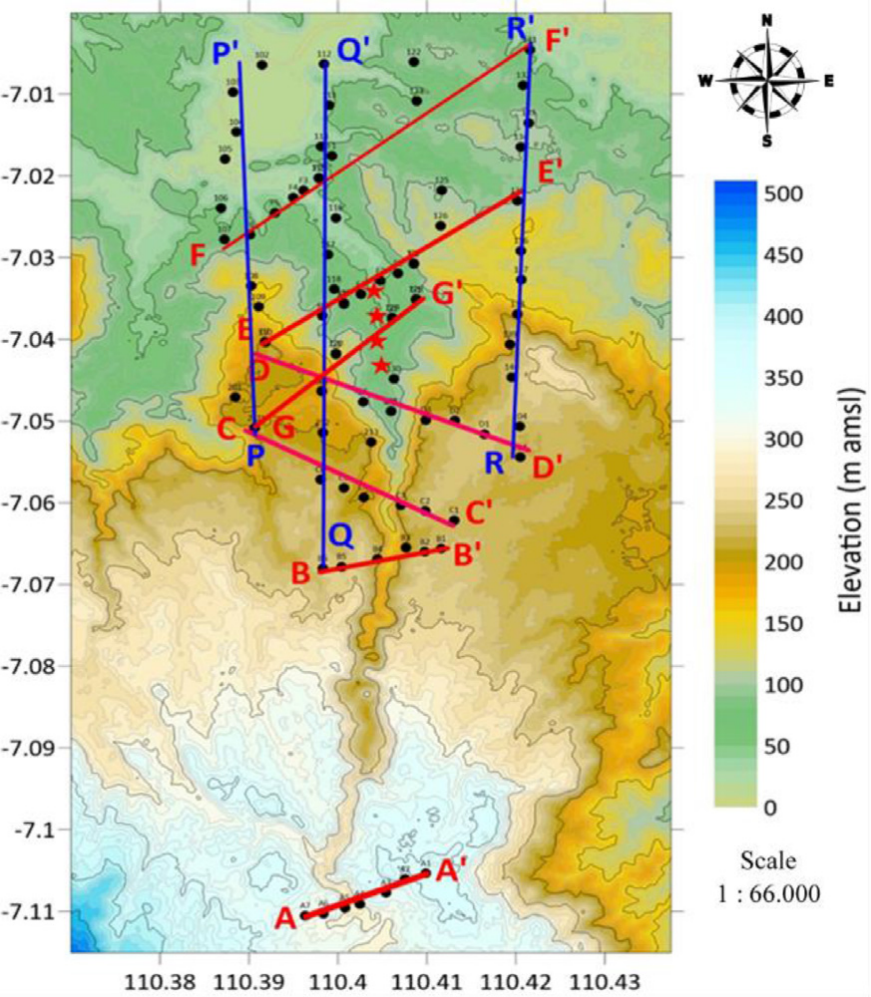
Acquisition of microtremor in 2D.

The Kaligarang Fault is a fault located in the city of Semarang in a north-south direction. According to Helmy [1] that the Kaligarang fault is a shear fault that has a relatively north-south direction (N5 E–N185 E). There are 7 fault locations in the Kaligarang main fault zone. This zone continues to develop until now so there is a development of restraining and realising [2]. Fig. 1 describes the location of data collection for a total of 75 points in the Kaligarang fault zone with 2D acquisition models. The vertical side in Fig. 1 explains Latitude and the Horizontal side explains Longitude. The star image in Fig. 1 explains the location of the chain structure. The height at each data point varies greatly, which is around 13 – 358 meters above sea level. Location data is written in the attachment coordinates.xlsx which can be opened via Ms. excell. The file in coordinate.xlsx provides information regarding the location of each point in the coordinate system: UTM degree and Longitude-Latitude

Research on the Kaligarang Fault zone at the study site was carried out using a microtremor and global positioning system (GPS). While the software used in data processing is Microsoft Excel, Notepad++, Geopsy for microtremor data, and Dinver, Surfer 13. The raw data on microtremor obtained is in the form of .txt which can be opened in the Notepad application. The data in the .txt describes the microtremor signal received by the seismograph and recorded by the data logger.

**Fig. 2:**
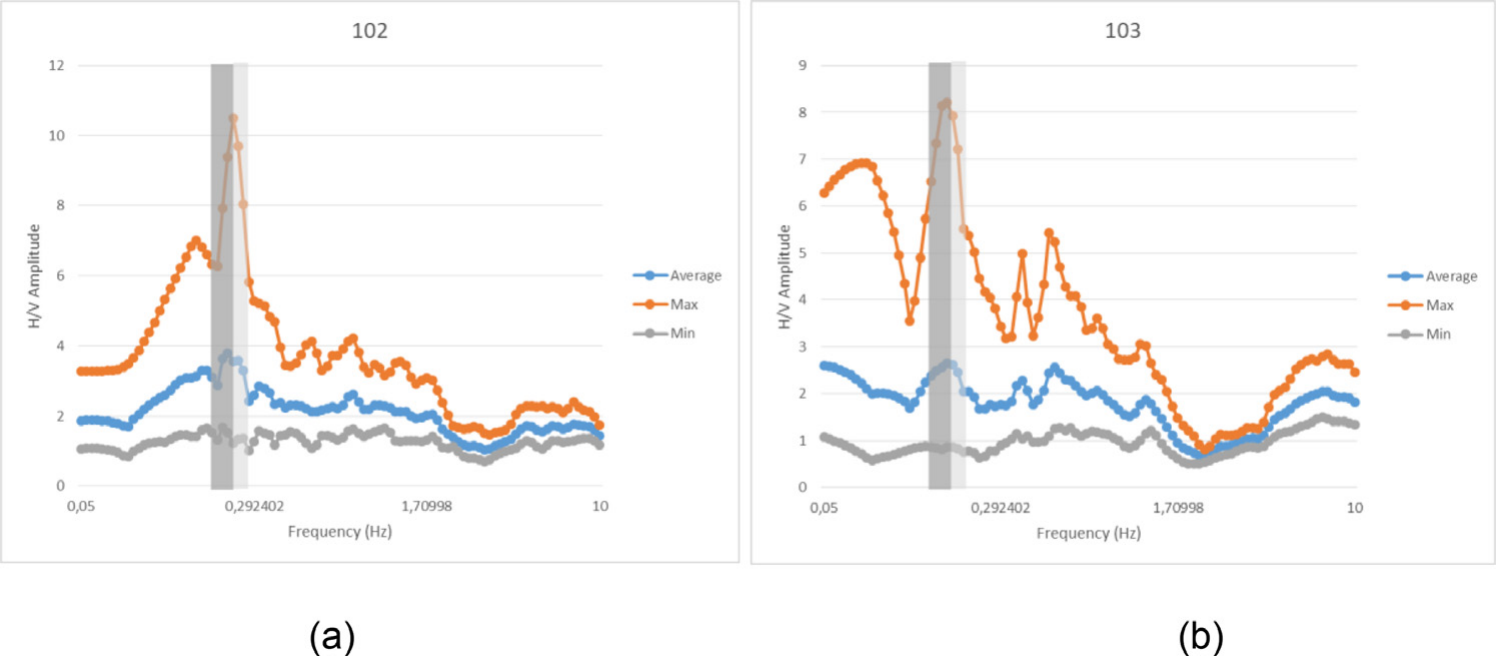
The HVSR curve on data (a) 102 (b) 103.

The raw data is then processed using the HVSR method. The sampling frequency used in processing HVSR data is 20 Hz. In addition, this study uses different tentative window lengths (such as 25, 30, 35, and 40 s) to obtain a proper and good HVSR curve. The results of the HVSR curve can be seen in Fig. 2 The results of data processing obtained in the processing of the HVSR method are HVSR curves in .hv format which can be opened using Notepad ++. The contents of the .hv format are the frequency, minimum, maximum and average values of the H/V Amplitude.

**Fig. 3:**
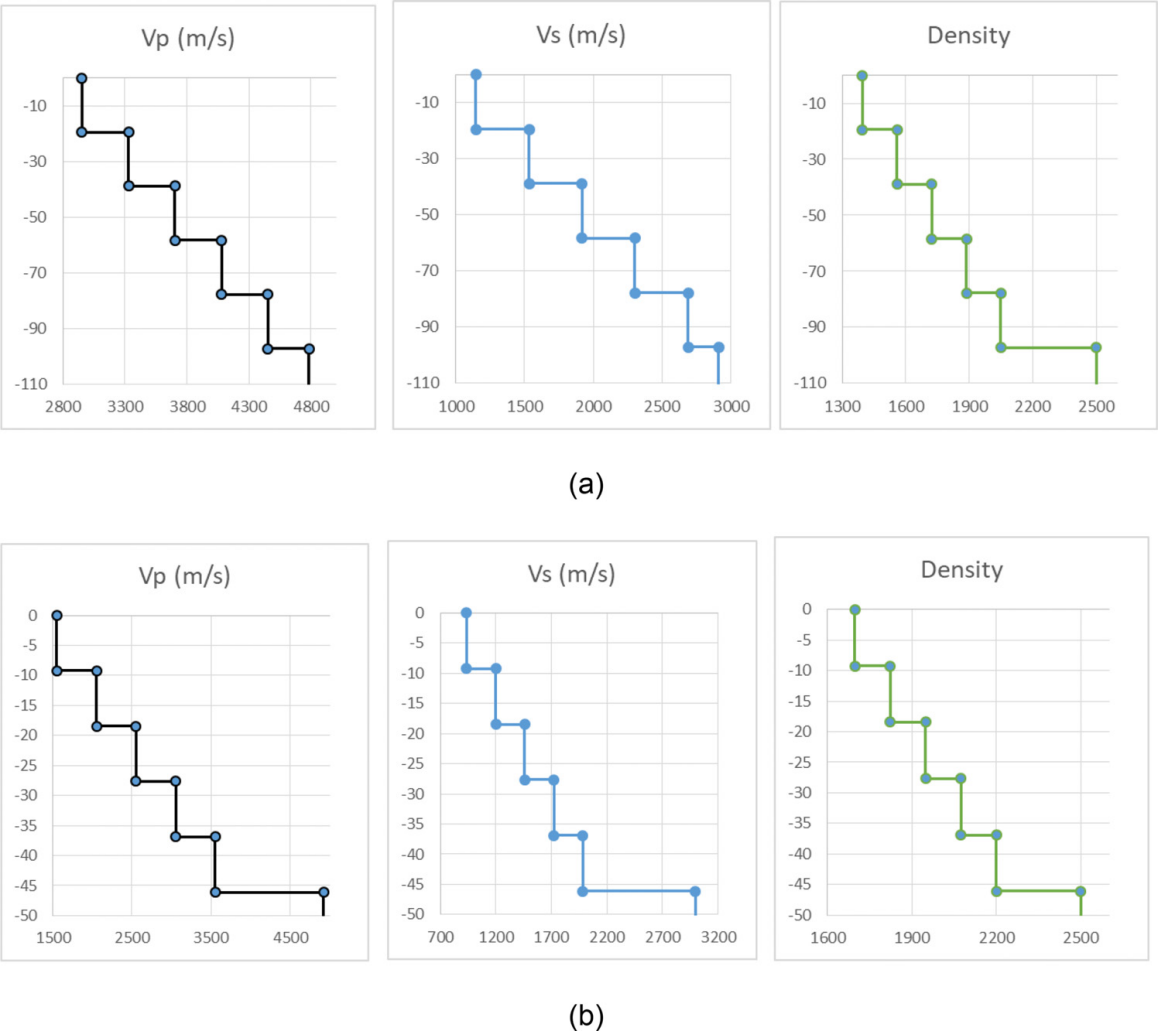
The inversion model on data (a) 102 (b) 103.

HV curve processing can be done using dinver software. The v_p_, v_s_, density, and thickness profiles of each data were obtained from carrying out the inversion process using dinver. The shape of the inversion results carried out by the diver software can be seen in Fig. 3. The result of the inversion is still in .model format and can be opened using Notepad++.

## 3 Experimental Design, Materials and Methods

### 3.1 HVSR Methods

The principle of the HVSR method is to compare the spectrum of the horizontal component with the vertical component of the spectrum of microwaves by assuming that most of the microwaves consist of shear waves and ignore surface waves (Rayleigh and Love waves). This method is almost the same as the transfer function between wave vibrations in sediment and bedrock. According to Nakamura [3] this method is an analytical method based on observations of shear wave propagation due to earthquake events for various geological conditions which can be estimated from the peak period of the microtremor H/V ratio [4]. In general, the HVSR method is a passive seismic method that uses three components in its measurement, namely 2 horizontal components East-West (East-West) and North-South (North-South), and 1 vertical component. Based on Herak [5], natural frequency and amplification are important parameters resulting from the HVSR method and can be used to determine local geological characterization. Ambient noise data can be used for HVSR and array analysis, which is important for obtaining the fundamental frequency of the location and the ellipticity of the fundamental mode of the Rayleigh wave at the location being measured. Array analysis is useful for obtaining dispersion curves, which are needed to estimate the shear wave velocity profile [6].

### 3.2 Processing Data

Data processing using the HVSR method was carried out with Geopsy software. The results of processing data using geopsy software are H/V curves and obtaining frequency (f_0_), period, and amplitude values at each data collection point [7]. Subsequent processing is carried out in the dinver software. Dinver software is an inversion application that functions to get density, v_p_, v_s_, and depth [8]. The values of density, v_p_, v_s_, and depth can be calculated via Ms. Excel to obtain other parameters [9].

## Ethic Statements

This work does not involve human subjects, animal experiments, or any data collected from social media platforms.

## CRediT authorship contribution statement

**Tony Yulianto:** Conceptualization, Methodology, Formal analysis, Supervision, Investigation, Writing – original draft, Writing – review & editing. **Gatot Yuliyanto:** Validation, Software, Formal analysis, Investigation, Data curation.

## Declaration of Competing Interest

The authors declare that they have no known competing financial interests or personal relationships that could have appeared to influence the work reported in this paper.

## Data Availability

Microtremor Data (Original data) (Mendeley Data).
